# Advantages and Challenges of Using Antimicrobial Peptides in Synergism with Antibiotics for Treating Multidrug-Resistant Bacteria

**DOI:** 10.1021/acsinfecdis.4c00702

**Published:** 2025-01-24

**Authors:** Regina Meneses Gonçalves, Bruna Estéfani Dutra Monges, Karen Garcia Nogueira Oshiro, Elizabete de Souza Cândido, João Pedro Farias Pimentel, Octávio Luiz Franco, Marlon Henrique Cardoso

**Affiliations:** † S-Inova Biotech, Programa de Pós-Graduação em Biotecnologia, Universidade Católica Dom Bosco, Campo Grande, MS 79117900, Brazil; ‡ Centro de Análises Proteômicas e Bioquímicas, Programa de Pós-Graduação em Ciências Genômicas e Biotecnologia, Universidade Católica de Brasília, Brasília, DF 71966700, Brazil; § Programa de Pós-Graduação em Ciências Ambientais e Sustentabilidade Agropecuária, Universidade Católica Dom Bosco, Campo Grande, MS 79117900, Brazil

**Keywords:** bacterial infections, antimicrobial peptides, antibiotics, synergism, combination therapy

## Abstract

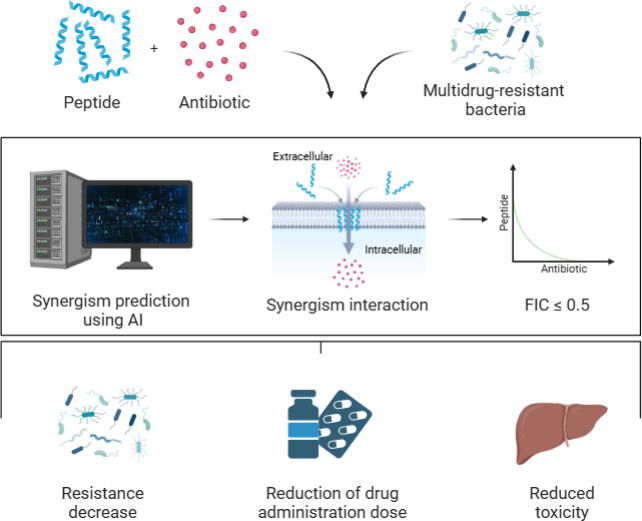

Multidrug-resistant bacteria (MDR) have become a global threat, impairing positive outcomes in many cases of infectious diseases. Treating bacterial infections with antibiotic monotherapy has become a huge challenge in modern medicine. Although conventional antibiotics can be efficient against many bacteria, there is still a need to develop antimicrobial agents that act against MDR bacteria. Bioactive peptides, particularly effective against specific types of bacteria, are recognized for their selective and effective action against microorganisms and, at the same time, are relatively safe and well tolerated. Therefore, a growing number of works have proposed the use of antimicrobial peptides (AMPs) in synergism with commercial antibiotics as an alternative therapeutic strategy. This review provides an overview of the critical parameters for using AMPs in synergism with antibiotics as well as addressing the strengths and weaknesses of this combination therapy using *in vitro* and *in vivo* models of infection. We also cover the challenges and perspectives of using this approach for clinical practice and the advantages of applying artificial intelligence strategies to predict the most promising combination therapies between AMPs and antibiotics.

## 1 Introduction

Bacterial infections treated with antibiotics and monotherapy represent one of the most significant challenges in modern medicine.^1^ Additionally, the World Health Organization (WHO) states that therapeutic options for treating infections are increasingly limited due to antibacterial resistance mechanisms, considerably increasing morbidity and mortality rates associated with infectious diseases caused by bacteria.^2^ This situation also alerts us to a scenario of increased bacterial resistance after the COVID-19 pandemic due to the indiscriminate use of antibiotics, which may increase the speed of dissemination of new resistance genes globally.^3^ In this context, multidrug-resistant (MDR) bacterial infections, characterized by different chemical structures and different mechanisms of action, may cause more deaths than chronic diseases, including cancer and diabetes.^1,4^ MDR bacteria are responsible for most nosocomial infections, and they often include the “ESKAPE” pathogens, *Enterococcus faecium*, *Staphylococcus aureus*, *Klebsiella pneumoniae*, *Acinetobacter baumannii*, *Pseudomonas aeruginosa*, and *Enterobacter* spp. Among them, *A. baumannii*, *P. aeruginosa*, and Enterobacteriaceae have been listed by the WHO as high- or medium-priority pathogens.^5^

When developing resistance to drugs, bacteria can adopt several mechanisms, including the production of β-lactamases, impermeability of the outer membrane in Gram-negative bacteria, efflux pumps, and modification of target proteins or receptors, among others.^6^ These resistance mechanisms can be acquired by mutations or through horizontal gene transfer, intensified due to exposure to different drugs and strong selective pressure; additionally, the genes that encode resistance can remain silent, and in the absence of antibiotics, resistance does not manifest.^7^ Thus, resistance mechanisms have resulted in the simultaneous development of bacterial resistance to numerous antibiotic classes, since bacteria are efficient in synthesizing and sharing genes, being capable of adapting to new niches and causing a broad spectrum of diseases.^5,8^ In this scenario, we observe that there is a progressive reduction in the effectiveness of conventional antibiotics against MDR bacterial infections associated with the limited number of new antimicrobials for bacterial treatment. Therefore, intensive studies are needed to develop new, nonconventional antibacterial drugs.^9^

Aiming at more effective therapies to treat MDR bacterial infections, different antimicrobial agents combined with conventional antibiotics have already been evaluated.^10^ Among them, we can highlight antimicrobial peptides (AMPs), since they have therapeutic potential against MDR bacteria.^11−13^ AMPs are considered a diverse group of bioactive molecules, 6 to 50 amino acid residues in length, with structural and functional diversity.^14^ Diverse cell types and tissues can produce this class of bioactive molecules in a variety of species, including plants, invertebrate animals, vertebrates, fungi, and bacteria.^15^ AMPs have some interesting characteristics, including a broad spectrum of antimicrobial activity and diverse mechanisms of action (e.g., membrane-associated mechanisms and intracellular targets).^16^

AMPs can act in different ways, for example, directly interrupting or causing damage to bacterial cell membranes, modulating the immune response, regulating inflammation, and also intracellular mechanisms.^16^ Additionally, these molecules have multifunctional activity profiles, presenting antibacterial, antifungal, and immunomodulatory activities.^15^ Some of these activities have revealed promising additive or synergistic action between AMPs and conventional antibiotics.^11,12,15^

Although some studies have reported the positive effects of AMP–antibiotic synergism for treating MDR bacterial infections, this combination therapy is often controversial. For example, some studies have shown that combination may or may not result in the elimination of a given bacterium *in vitro* or *in vivo*.^12,14^ In this regard, a crucial question stands out: how successful has AMP–antibiotic combination therapy been and what are the determinants for positive anti-infective effects? Bearing this in mind, we investigated the subjects that permeate these questions and provide an overview of AMP–antibiotic synergism as a promising therapeutic strategy.

## 2 Differences in Effectiveness between Monotherapy, Combination Therapy, and Synergism

It is known that approximately half of the antibiotics currently used were discovered between the 1950s and 1960s. Nevertheless, the efficiency of these drugs decreased as bacterial resistance evolved and spread.^17,18^ Some factors significantly contributed to a drastic decrease in antibiotic discovery and their translation to the clinic over the following years. Among them, we can mention the lack of interest from pharmaceutical companies due to the low return on investment, in addition to the fact that the research process as a whole is difficult and time-consuming.^17,19^

The practice of drug association has been a reality since the beginning of the antibiotics era,^20^ where combination therapies allowed the reduction of the drug administration dose without altering the antimicrobial activity and, consequently, could promote the reduction of toxic effects.^21^

### 2.1 Monotherapy

The first synthetic antibiotic was developed in 1910, salvarsan (based on arsenic), used to treat *Treponema pallidum*, the causative agent of syphilis.^22^ Later, salvarsan was replaced by sulfonamides, the first class of broad-spectrum antimicrobials with clinical efficacy, used until today, which were also replaced by the discovery of penicillin.^22^

Penicillin was discovered in 1928, and within a year after its first clinical use in 1941, cases of penicillin-resistant staphylococci emerged, revealing that bacteria had long been one step ahead.^23,24^ For years, the development and subsequent clinical use of antibiotics to treat bacterial infections have primarily been driven by monotherapy.^23^ It is known that since the mid-20th century, with the approval of the first antibiotics, there has been a drastic slowdown in the development of new antimicrobials against bacterial pathogens.^25^ Most are chemically modified variants of already approved antibiotics, many of which are derived from natural products.^25^

Some studies recommend monotherapy, for example, to treat hospital-acquired pneumonia.^26^ However, monotherapy can only be indicated without risk factors for MDR bacteria.^26^ Additionally, some antibiotics are often reserved as a last resort for treating bacterial infections, such as colistin and polymyxin B for Gram-negative bacteria and daptomycin for Gram-positive bacteria.^24,27^

Currently, antibiotic monotherapy, such as colistin, is still used against bloodstream infections caused by *Klebsiella pneumoniae*.^28^ However, colistin is considered a last-line antimicrobial agent, and its use must be cautious, as this antibiotic may increase the rate of nephrotoxicity when compared to β-lactams.^28^ Another example in which monotherapy is recommended, including by the Infectious Disease Society of America (IDSA), is in treating Lyme disease.^29^ In this case, monotherapy with doxycycline or amoxicillin can be effective from 3 to 30 days after the tick bite, with up to 89% response to the treatment.^29^

Although antibiotics have profoundly contributed to human and animal health, their indiscriminate use has resulted in MDR, which is extremely difficult to combat, making monotherapy ineffective and challenging.^30^ Fighting bacterial infections with antibiotic monotherapy represents the first solution. However, there are great difficulties in this regard, making the development of new therapies extremely urgent.^31^

### 2.2 Combination Therapy and Synergism

Combination therapy has become standard due to the many benefits it offers over monotherapy.^32,33^ This type of therapy allows the use of smaller doses of drugs, enhancing their beneficial effects and reducing adverse effects, which can also have a synergistic effect against a specific target.^34^ Since bacterial resistance is related to the time of exposure to antibiotics, there is a need for effective therapies that have a broad spectrum of action and rapid kinetics of death. Combination therapy can meet these requirements.^35^ Combination therapy approaches broaden the spectrum of susceptible pathogens and can help manage polymicrobial infections when two or more different classes of antimicrobial agents are needed to kill the pathogens.^32^ Another potential benefit of this type of therapy is that it can delay or even prevent the emergence of resistance among pathogens since the chances of developing resistance to two drugs are lower than that with a single drug.^36^ For example, if two drugs are administrated in combination, the first drug could eradicate the strain resistant to the second drug and vice versa, thus avoiding resistance to both drugs.^32^

Diverse authors mention that it is still controversial whether combination antimicrobial therapy is more effective than monotherapy for Gram-negative bacterial infections.^37,38^ This is because there may be possible disadvantages such as antagonism, superinfection, increased incidence of adverse effects, and increased cost, which must be considered.^11^ However, combination therapy is particularly recommended in clinical practice to treat life-threatening infections when an antimicrobial is not broad spectrum and the infection is polymicrobial.^11,36,38^ Antibiotic combinations are applied in up to 50% of patient cases in the treatment of severe surgical site infections, bacteremia, pneumonia, or septic shock.^23^ The use of combination therapy for Gram-negative bacterial infections can generally be justified for the following reasons: (i) to prevent or delay the emergence of resistance during antimicrobial therapy,^39^ (ii) to broaden the empirical coverage provided by two antimicrobial agents with different spectra of activity (an effort to ensure that the pathogen is adequately covered by at least one of the two components of the regimen),^40^ or (iii) to explore the synergy observed *in vitro* between two antibiotic agents compared to just one and, thus, improve clinical outcomes.^41^

For a long time, studies have demonstrated the effectiveness of combination therapy to treat *Helicobacter pylori* infections^42^ or even *Mycobacterium tuberculosis* infections.^43^ Prolonged combination therapies are often employed to treat bacterial infections related to endocarditis.^44^ Another example is the potentiation of vancomycin’s antibacterial effects against *Escherichia coli* combined with trimethoprim or nitrofurantoin.^45^

**Figure 1:**
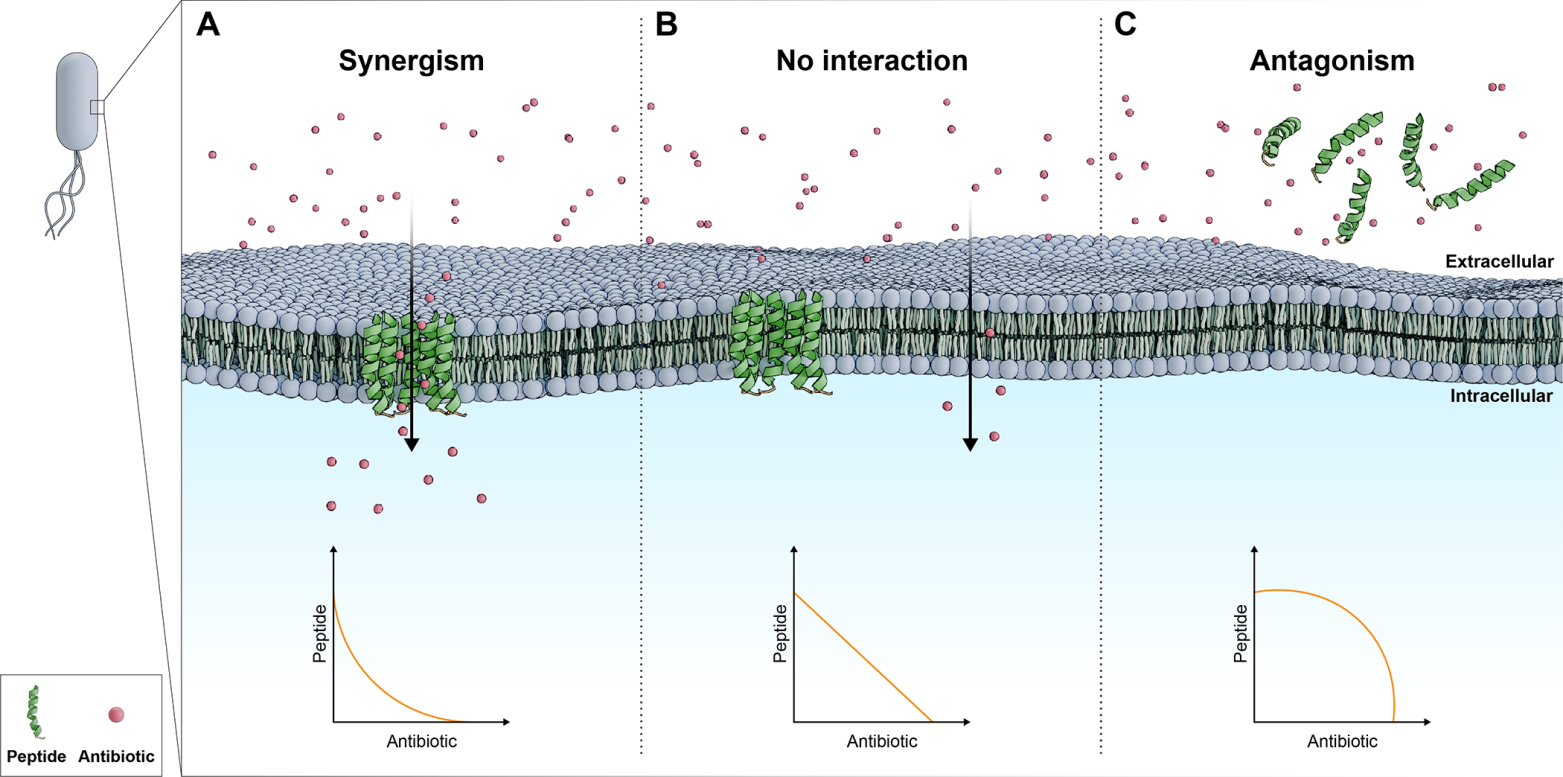
Examples of synergism. (A) Synergism: Peptides and antibiotics act together, requiring a lower concentration to kill bacteria. (B) No interaction: Peptides and antibiotics act independently, leaving the required concentration unchanged. (C) Antagonism: Peptides and antibiotics can interact with each other, requiring a higher concentration to kill bacteria effectively.

Studies reporting combination therapies have classified interactions between various agents as antagonistic or synergistic. When the inhibitory effects are less than the additive effect of each drug individually, it is known as antagonism.^34^ When the combination of two compounds exerts inhibitory effects that are more significant than the sum of the effects of each alone, we can say that synergism occurs.^11,34^ To determine the antimicrobial synergy, the checkerboard assay is most used, where the fractional inhibitory concentration index (FICI) is established, with synergy defined by FICI ≤ 0.5, no interaction defined by FICI = 0.5–4.0 and antagonism defined by FICI > 4.0 (Figure 1).^30^

**Figure 2:**
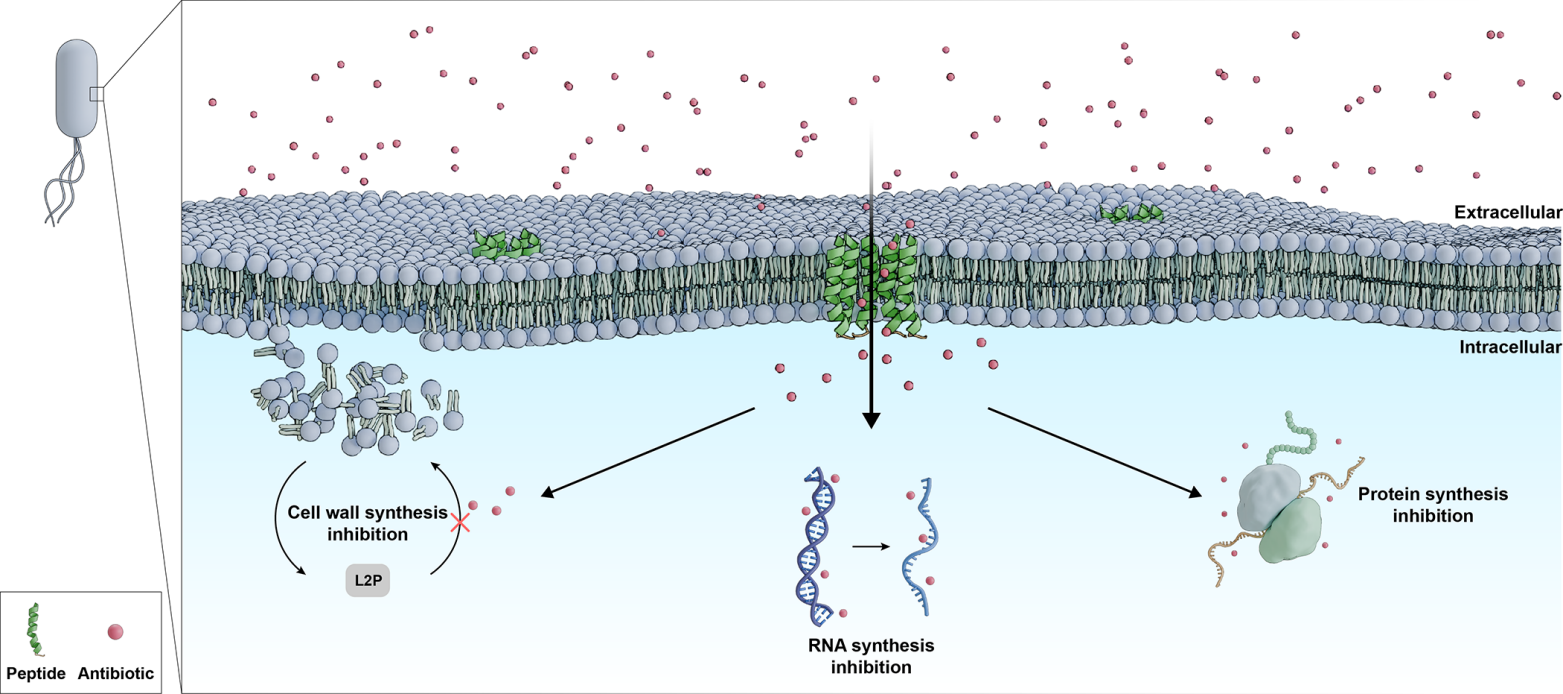
Synergistic mechanism between AMPs and antibiotics against Gram-negative bacteria involves the disruption of bacterial membranes by the peptides, enabling enhanced antibiotic entry. The antibiotics in the figure (e.g., imipenem, meropenem, rifampicin, and azithromycin) target intracellular processes, including cell wall synthesis, RNA synthesis inhibition, and protein synthesis inhibition.

AMP–antibiotic synergism is justified by the fact that the mode of action of AMPs, in most cases, is to reach the bacterial membrane, destabilizing it or forming pores.^46^ This favors the entry of antibiotics into the bacteria, which in general have intracellular mechanisms of action, such as inhibition of DNA replication, DNA transcription, or cell wall synthesis.^11,46^ These characteristics can facilitate and promote synergism, as different biological targets are affected (Figure 2).^46^

In recent years, many researchers have focused on combination therapies between AMPs and antibiotics, as AMPs have bright prospects as new therapeutic agents.^30,47^ Bearing this in mind, we can cite several examples of AMPs in synergy with antibiotics, including the FK16 peptide (Table 1), derived from LL-37, which showed synergistic activity with vancomycin *in vitro* against three strains of *P. aeruginosa* (Table 2).^48^ Another study reports the *in vitro* synergism of four tryptophan-containing peptides L11W, L12W, I1WL5W, and I4WL5W (Table 1), with commercially available antibiotics, including penicillin, ampicillin, erythromycin, and tetracycline.^49^ The combination of these peptides with at least three of these antibiotics resulted in satisfactory activities against multidrug-resistant *Staphylococcus epidermidis* (Table 2).^49^

**Table 1:** Sequences and Origin of AMPs That Have Been Used in Synergy with Conventional Antibiotics^a^

peptides	sequences	origin	refs
FK16	FKRIVQRIKDFLRNLV	FK16 is a cathelicidin (LL-37)-derived peptide	(48)
L11W	IKKILSKIKKWLK-NH_2_	L11W is derived from the frog skin peptide temporin-1CEb	(49)
L12W	IKKILSKIKKLWK-NH_2_	L12W is derived from the frog skin peptide temporin-1CEb	(49)
I1WL5W	WKKIWSKIKKLLK-NH_2_	I1WL5W is derived from the frog skin peptide temporin-1CEb	(49)
I4WL5W	IKKWWSKIKKLLK-NH_2_	I4WL5W is derived from the frog skin peptide temporin-1CEb	(49)
Sphistin	AGGKAGKDSGKSKAKAVSRSARAGLQFPVGRIHRHLK	Sphistin is derived from the mud crab *Scylla paramamosain*	(50)
Sph_12–38_	KAKAKAVSRSARAGLQFPVGRIHRHLK	Sph_12–38_ is a truncated short fragment from Sphistin	(50)
Esc(1–21)	GIFSKLAGKKIKNLLISGLKG-NH_2_	Esc(1–21) is derived from esculentin-1a	(36)
BP203	KKLFKKILRYL-NH_2_	BP203 is a BP100 analog derived from a cecropin A–melittin hybrid	(51)
MAP-0403 J-2	KWLRRPWRRWR-NH_2_	MAP-0403 J-2 is a MAP-0403 analog, derived from Ixosin-B, an AMP isolated from the salivary glands of the hard tick *Ixodes sinensis*	(51)
A3-APO	(H-Chex-RPDKPRPYLPRPRPPRPVR)_2_-Dab-NH_2_	A3-APO is a dimer designed *de novo* starting from a sequence comparison of different insect-derived PrAMPs	(47)
Chex1-Arg20	(H-Chex-RPDKPRPYLPRPPPRPVR-NH_2_	ARV-1502^b^ was designed *de novo*, starting from a sequence comparison of different insect-derived PrAMPs	(47)
Tridecaptin M	G-d-Dab-G-d-S-d-W-S-Dab-d-Dab-I-E-I-d-αI-S	Tridecaptin M is derived from mud bacterium	(52)
Esc(1–21)-1c	GIFSKLAGKKIKN*l*LI*s*GLKG-NH_2_^c^	Esc(1–21)-1c is derived from esculentin-1a	(53)

a Abbreviations: Chex, 1-amino-cyclohexane carboxylic acid; Dab, 2,4-diamino-butyric acid; PrAMPs, proline-rich antimicrobial peptides.

b ARV-1502 is a commercial name.

c The d-amino acids at positions 14 and 17 are shown in italics.

**Table 2:** Synergism Strategies Involving AMPs and Antibiotics

peptide	antibiotic	bacteria	types of activity and experimental assays^a^	proposed mechanisms of action (AMP; antibiotic)	ref
FK16	vancomycin	*P. aeruginosa* PAO1	synergism (FICI = 0.25) *in vitro* tests	membrane rupture; inhibition of cell wall synthesis	(48)
		*P. aeruginosa* ATCC 19660	synergism (FICI = 0.37) *in vitro* tests		
		*P. aeruginosa* OS	synergism (FICI = 0.37) *in vitro* tests		
L11W	penicillin	multidrug-resistant *S. epidermidis*	synergism (FICI = 0.31) *in vitro* tests	membrane disruption; inhibition of cell wall synthesis	(49)
	ampicillin		synergism (FICI = 0.28) *in vitro* tests	membrane disruption; inhibition of cell wall synthesis	
	erythromycin		synergism (FICI = 0.28) *in vitro* tests	membrane disruption; binding to 50S ribosomal subunits, blocking protein synthesis	
L12W	penicillin	multidrug-resistant *S. epidermidis*	synergism (FICI = 0.28) *in vitro* tests	membrane disruption; inhibition of cell wall synthesis	(49)
	ampicillin		synergism (FICI = 0.25) *in vitro* tests	membrane disruption; inhibition of cell wall synthesis	
	erythromycin		synergism (FICI = 0.28) *in vitro* tests	membrane disruption; binding to 50S ribosomal subunits, blocking protein synthesis	
I1WL5W	penicillin	multidrug-resistant *S. epidermidis*	synergism (FICI = 0.28) *in vitro* tests	membrane disruption; inhibition of cell wall synthesis	(49)
	ampicillin		synergism (FICI = 0.25) *in vitro* tests	membrane disruption; inhibition of cell wall synthesis	
	erythromycin		synergism (FICI = 0.28) *in vitro* tests	membrane disruption; binding to 50S ribosomal subunits, blocking protein synthesis	
	tetracycline		synergism (FICI = 0.28) *in vitro* tests	membrane disruption; binding to 30S ribosomal subunits, blocking protein synthesis	
I4WL5W	penicillin	multidrug-resistant *S. epidermidis*	synergism (FICI = 0.18) *in vitro* tests	membrane disruption; inhibition of cell wall synthesis	(49)
	ampicillin		synergism (FICI = 0.15) *in vitro* tests	membrane disruption; inhibition of cell wall synthesis	
	erythromycin		synergism (FICI = 0.31) *in vitro* tests	membrane disruption; binding to 50S ribosomal subunits, blocking protein synthesis	
Sphistin	rifampicin	*P. aeruginosa* ATCC 9027	synergism (FICI = 0.31) *in vitro* tests	membrane disruption; inhibition of RNA synthesis	(50)
	azithromycin		synergism (FICI = 0.31) *in vitro* tests	membrane disruption; inhibition of protein synthesis	
Sph_12–38_	rifampicin	*P. aeruginosa* ATCC 9027	synergism (FICI = 0.37) *in vitro* tests; complete wound healing *in vivo* model between 5 and 7 days	membrane disruption; inhibition of RNA synthesis	(50)
	azithromycin		synergism (FICI = 0.22) *in vitro* tests; complete wound healing *in vivo* model between 4 and 5 days	membrane disruption; inhibition of protein synthesis	
Esc(1–21)	colistin	*A. baumannii* (four distinct strains)	synergism (FICI = 0.25–0.37) *in vitro* tests	membrane perturbation in both	(36, 54)
BP203	rifampicin	colistin-resistant *E. coli*	synergism (FICI = 0.31–0.50) *in vitro* tests	b; inhibition of cell wall synthesis	(51, 55)
	meropenem	colistin-resistant *K. pneumoniae*	synergism (FICI = 0.25–0.50) *in vitro* tests	b; inhibition of cell wall synthesis	
	chloramphenicol		synergism (FICI = 0.14–0.37) *in vitro* tests	b; inhibition of protein synthesis	
	rifampicin		synergism (FICI = 0.02–0.31) *in vitro* tests	b; inhibition of RNA synthesis	
	ciprofloxacin		synergism (FICI = 0.37) *in vitro* tests	b; inhibition of type II topoisomerase (DNA gyrase) and topoisomerase IV	
	ceftazidime		synergism (FICI = 0.07–0.18) *in vitro* tests	b; inhibition of cell wall synthesis	
MAP-0403 J-2	colistin	colistin-resistant *E. coli*	synergism (FICI = 0.31–0.50) *in vitro* tests	b; membrane perturbation	(51, 56)
		chloramphenicol	synergism (FICI = 0.50) *in vitro* tests	b; inhibition of protein synthesis	
		rifampicin	synergism (FICI = 0.18–0.50) *in vitro* tests	b; inhibition of RNA synthesis	
		ceftazidime	synergism (FICI = 0.25–0.37) *in vitro* tests	b; inhibition of cell wall synthesis	
	chloramphenicol	colistin-resistant *K. pneumoniae*	synergism (FICI = 0.25–0.37) *in vitro* tests	b; inhibition of protein synthesis	
		rifampicin	synergism (FICI = 0.07–0.37) *in vitro* tests	b; inhibition of RNA synthesis	
		ciprofloxacin	synergism (FICI = 0.37) *in vitro* tests	b; inhibition of type II topoisomerase (DNA gyrase) and topoisomerase IV	
		ceftazidime	synergism (FICI = 0.12–0.50) *in vitro* tests	b; inhibition of cell wall synthesis	
A3-APO	colistin	*K. pneumoniae* K97/09	synergism (FICI = 0.08) *in vitro* tests; increased survival rate by 100% *in vivo* tests	disintegrates bacterial membrane and inhibits the 70 kDa heat shock protein DnaK; membrane perturbation	(47, 57)
	imipenem	*A. baumannii* BAA-1605	synergism (FICI = 0.08) *in vitro* tests	disintegrates bacterial membrane and inhibits the 70 kDa heat shock protein DnaK; inhibition of cell wall synthesis	
Chex1-Arg20^c^	meropenem	*E. coli* UNT167-1	synergism (FICI = 0.38) *in vitro* tests	membrane rupture; inhibition of cell wall synthesis	(47, 58)
	ceftazidime	*Burkholderia pseudomallei* 1026b	increased survival rate by 80% *in vivo* tests	membrane rupture; inhibition of cell wall synthesis	
Tridecaptin M	rifampicin	*A. baumannii* ATCC 19606	synergism (FICI = 0.31) *in vitro* tests	membrane permeability; inhibition of RNA synthesis	(52, 59)
	vancomycin		synergism (FICI = 0.31) *in vitro* tests	membrane permeability; inhibition of cell wall synthesis	
	clarithromycin		synergism (FICI = 0.31) *in vitro* tests	membrane permeability; binding to 50S ribosomal subunits, blocking protein synthesis	
	imipenem		synergism (FICI = 0.37) *in vitro* tests	membrane permeability; inhibition of cell wall synthesis	
	ceftazidime		synergism (FICI = 0.28) *in vitro* tests	membrane permeability; inhibition of cell wall synthesis	
	ceftazidime	*A. baumannii* ATCC 2803	synergism (FICI = 0.31) *in vitro* tests	membrane permeability; inhibition of cell wall synthesis	
	rifampicin	*A. baumannii* AB1	synergism (FICI = 0.27) *in vitro* tests	membrane permeability; inhibition of RNA synthesis	
	vancomycin		synergism (FICI = 0.31) *in vitro* tests	membrane permeability; inhibition of cell wall synthesis	
	rifampicin	*A. baumannii* AB2	synergism (FICI = 0.28) *in vitro* tests	membrane permeability; inhibition of RNA synthesis	
	vancomycin		synergism (FICI = 0.31) *in vitro* tests	membrane permeability; inhibition of cell wall synthesis	
	rifampicin	*A. baumannii* GMCH05	synergism (FICI = 0.25) *in vitro* tests	membrane permeability; inhibition of RNA synthesis	
	vancomycin		synergism (FICI = 0.28) *in vitro* tests	membrane permeability; inhibition of cell wall synthesis	
	ceftazidime		synergism (FICI = 0.28) *in vitro* tests	membrane permeability; inhibition of cell wall synthesis	
	rifampicin	*A. baumannii* ATCC 19606	bacterial death below the detection limit in 4 h in an *ex vivo* blood infection model	membrane permeability; inhibition of RNA synthesis	
Esc(1–21)-1c	erythromycin	*P. aeruginosa* PAO1	synergism (FICI = 0.37) *in vitro* tests	membrane perturbation; binding to 50S ribosomal subunits, blocking protein synthesis	(53, 54)
	chloramphenicol		synergism (FICI = 0.25) *in vitro* tests	membrane perturbation; inhibition of protein synthesis	
	tetracycline		synergism (FICI = 0.37) *in vitro* tests	membrane perturbation; binding to 30S ribosomal subunits, blocking protein synthesis	

a FICI, Fractional Inhibitory Concentration Index.

b Not reported.

c ARV-1502 is the commercial name.

Studies also revealed the antimicrobial efficacy of two AMPs, called Sphistin and Sph_12–38_ (Table 1), combined with the antibiotics rifampicin and azithromycin against *P. aeruginosa*, where synergistic activity was observed *in vitro* (Table 2).^50^ Furthermore, in that same work, the combinations were evaluated in an *in vivo* wound model, promoting complete healing in less time when compared to the controls.^50^ It has also been reported that *A. baumannii* strains can be combatted with synergism, where the Esc(1–21) peptide (Table 1) along with colistin acted together to inhibit growth and kill four clinical isolates of this bacterium (Table 2).^36^

Two peptides, BP203 and MAP-0403 J-2 (Table 1), in combination with six antibiotics (e.g., colistin, meropenem, chloramphenicol, rifampicin, ciprofloxacin, and ceftazidime), showed interesting results when used against *E. coli* and *K. pneumoniae* strains resistant to colistin.^51^ BP203 was more effective against colistin-resistant *K. pneumoniae* in different antibiotic combinations. By contrast, only one combination was effective for colistin-resistant *E. coli* (Table 2). MAP-0403 J-2 showed synergism against both strains in at least four antibiotic combinations (Table 2).^51^

In addition to the examples mentioned above, some studies evaluated the synergism between antibiotics and peptides with chemical modifications in their sequences, which are called peptidomimetics. For example, some proline-rich AMPs (A3-APO and Chex1-Arg20) (Table 1) showed exciting results in synergism with imipenem, colistin, meropenem, and ceftazidime.^47^ These combinations resulted in satisfactory activities both *in vitro* and *in vivo* against antibiotic-resistant pathogens such as *K. pneumoniae*, *E. coli*, *A. baumannii*, and *Burkholderia pseudomallei* (Table 2).^47^

Combination of the tridecaptin M peptide (Table 1) with antibiotics such as rifampicin, vancomycin, clarithromycin, imipenem, and ceftazidime has also been used to treat different *A. baumannii* strains.^52^ Improved activity was observed when compared to treatment with antibiotic alone, as monotherapy, in addition to an *ex vivo* blood infection model showing complete bacterial killing using a combination of tridecaptin M and rifampicin (Table 2).^52^

Another study also reported the susceptibility of *P. aeruginosa* when challenged with the Esc(1–21)-1c peptide (Table 1) combined with different antibiotics.^53^*In vitro* synergistic activity of the Esc(1–21)-1c peptide was observed with at least three of the five antibiotics tested (Table 2).^53^

Given the above, it is observed that the combined use of AMP–antibiotics can provide many benefits, including improvement of the therapeutic effect of antibiotics and the reduction of their dosages, decreased AMP toxicity, as well as a lower propensity to trigger bacterial resistance.^30^

## 3 Challenges and Advantages of Using AMP–Antibiotic Synergism for Translational Purposes

While there are examples of effective synergistic combinations between AMPs and conventional antibiotics, some studies are controversial. The highly different or, in some cases, similar mechanisms of action for AMPs and antibiotics seem insufficient for synergistic activity.^11^ The methodology used to determine synergy, for instance, is a significant point when discrepancies appear. Most studies use the checkerboard experiment to evaluate the synergism between AMPs and antibiotics. This methodology consists of multiple dilutions of those two antimicrobials in microtiter plates, until the highest antibacterial activity at lower AMP/antibiotic doses is reached. Additionally, the checkerboard experiment is commonly used as the basis for the calculation of a fractional inhibitory concentration index (FICI), which is also prone to reproducibility problems.^60^

Time-kill experiments can determine if a combination is synergistic and whether it is bactericidal, and they provide data on bacteria killing over time. The bacteria are incubated with the antibiotics of interest, together or individually, and sampled at intervals throughout 24 h for quantitative culture. Moreover, it could be considered the gold standard for synergism evaluation, as it allows a dynamic assessment and higher sensitivity than other methods.^61^ Nevertheless, it is worth noting that determining synergy from dose–response curves can be quite challenging, mainly because they are not linear.

Despite the limitations cited above, both the checkerboard array and time-kill synergy *in vitro* methods can provide valuable information on drug combination activity and are particularly useful in evaluating novel therapeutic options for MDR bacteria treatment.^62^

Another point that must be considered is that the *in vitro* estimation of AMP and antibiotic activities may not reflect their *in vivo* efficacy.^50^ Additionally, studies have suggested that the observed synergy is not universal for combating bacterial strains and species with different resistance profiles *in vitro* and *in vivo*.^63^ For example, a study highlighted divergencies between the *in vitro* and *in vivo* activities of colistin in combination with meropenem against carbapenem-resistant *A. baumannii*.^64^ Although the combination of colistin and meropenem is synergistic *in vitro*, there was no significant improvement in the *in vivo* assays compared to colistin monotherapy.

There are many reasons why *in vitro* assays may not reflect *in vivo* activities, leading to a lack of success when transitioning from the biologically controlled environment to clinical practice.^34,65^ Reproducing general physiological conditions in the laboratory is challenging, and failures may include differences in local pH or salt concentration, nutrient distribution, or osmotic stress.^34,65^ Moreover, when it comes to animal models, it is expected that AMPs and antibiotics present different bioavailability and, consequently, different concentrations at the site of infection, which can impair their synergistic effect.^65^

Combination therapy must be balanced against possible disadvantages, such as antagonism, superinfection, increased incidence of adverse effects, and increased cost. In clinical practice, synergism is widely used in potentially fatal infections to target all pathogens when a single antimicrobial does not have a broad spectrum of action. Sometimes, the choice of combination therapy is made to prevent the emergence of resistance or when there is a polymicrobial infection untreatable with a single drug.^64,66^

AMPs have not always boosted the activities of antibiotics, resulting in a synergistic effect.^67^ Some challenges have emerged to translate this therapeutic strategy to the clinic. Despite the alarming increase in antimicrobial resistance, most pharmaceutical companies have abandoned the development of new antibiotics and have instead focused on developing more profitable drugs to treat noncommunicable diseases.^50^

Attempts to identify and design clinically relevant AMPs have led to thousands of molecules being identified, out of which only a few have proceeded to preclinical studies and clinical trials, including histatin-1 and -3, which are under phase I clinical trials to treat chronic *P. aeruginosa* infections (DRAMP18062).^50^ Interestingly, some studies have concluded that most trials have not been successful, mainly because of poorly designed experiments, low AMP bioavailability, inability to meet clinical effectiveness targets, and the absence of improved activity over conventional antibiotics.^64^

From the pharmaceutical point of view, adverse effects in drug combinations are caused by interactions of pharmacokinetics and pharmacodynamics.^68^ Pharmacodynamic interactions occur when medications directly influence each other’s effects, and these may be synergistic or antagonistic.^68^ These interactions can also occur outside the target bacteria, leading to unwanted effects on the host.^9,68^ By contrast, pharmacokinetics affect the absorption, distribution, metabolism, and elimination of drugs, leading to alterations in effective concentrations in the blood and tissue. This is important because exposure to subinhibitory concentrations of antibiotics accelerates the emergence and positive selection of resistant bacterial strains.^9,68^

Although AMPs have shown a broad spectrum of antibacterial activity *in vitro*, some have also resulted in systemic and local toxicity, hindering the successful transition from the bench to the clinic.^69^ AMP instability *in vivo* and their multiple activities that are not always expected in the host (e.g., pro-inflammatory and anti-inflammatory effects) still appear as substantial obstacles that must be overcome for translational medicine in synergistic therapy.^68^

The stability and permeability of AMPs can be considered one of the biggest challenges in clinical practice, as well as proteolytic degradation.^35,70^ However, it is already known that there are some ways to overcome this situation. An example of this is nanostructures, which can stabilize AMPs and ensure their interaction with the target.^70,71^ Nanostructures, such as nanoparticles, nanorods, nanowires, and 2D materials, can be an alternative for the transport and delivery of AMPs and medicines, which has already been mentioned in some studies.^70−72^ Furthermore, hydrogels, polymers, and electrospun fibers in conjunction with AMPs, recombinant genetic engineering strategies, and chemical modification of AMPs can also help to overcome the various limitations presented by AMPs, helping to improve their activity.^73^ Other examples that can increase the functionality of AMPs are peptidomimetic approaches that include glycosylation (interferes with the rigidity of AMPs), cyclization, and stapling (promotes stabilization of the structure while preserving the bioactive conformation), and also d-amino acids (which are more resistant to proteolysis).^74^

Despite the challenges faced in the practice of AMP–antibiotic synergism, we can also mention many advantages of using this therapy. The vast majority of AMPs have membrane-rupturing activity, which provides greater bioavailability of antibiotics when accessing the bacterial cell, improving the effectiveness of antimicrobials, in addition to minimizing the emergence of resistance.^49^ Reports from previous work show that the toxicity of AMPs can be reduced when used in synergism with rifampicin during the treatment of *Mycobacterium* infections, in addition to delaying the emergence of resistance to rifampicin.^75^

Another point that we must consider about the development of resistance is the bacterial exposure time to antibiotics. Therefore, in addition to achieving effective therapies, it is necessary to have drugs with a broad spectrum of action and rapid death kinetics.^35^ We find these requirements mainly in combination therapies, where different medications are used to treat a given disease, which provides more potent effects than single medications.^35,76^

Therefore, continued efforts to fill the gaps regarding AMP–antibiotic synergism are significant, as this therapy has attracted interest from researchers in the past decade and could become a promising strategy to combat MDR bacterial infections.^77^

## 4 Machine Learning for Predicting Combination Therapies

As an attempt to overcome some obstacles faced by AMP treatment, an interesting way to evaluate synergism and provide more assertive therapy is by using computational methods, which can predict drug combinations, helping to determine synergism.^34^ Before the experimental stage, these methods would facilitate the understanding of the likely drugs that would act synergistically, and after the experimental stage, computational methods would provide researchers with statistical data that would quantify the presence or absence of synergism.^34^

Artificial intelligence (AI) learning models have played a crucial role in the discovery and prediction of novel antimicrobial therapies, significantly facilitating the process and reducing associated costs and efforts.^78^ In the combination therapy context, machine learning (ML) models have also been successfully applied to predicting unknown interaction outcomes.^79^

Different ML models are used to predict combination interactions, depending on the input data provided and the desired output.^79^ The input data are crucial in determining the appropriate learning model. Labeled data, validated through experiments, can be used for supervised learning. For instance, molecules with confirmed synergistic or antagonistic interactions can train the algorithm. Conversely, unsupervised models can identify correlations within unlabeled data as they do not require confirmed outcomes to make predictions. In some cases, a hybrid approach can be employed, combining labeled and unlabeled data to enhance performance, especially when labeled data is scarce.^79,80^

The output data can primarily be categorized as either classification or regression. In a classification problem, outcomes are divided into distinct categories, including “synergistic” or “nonsynergistic”. By contrast, a regression problem involves predicting a continuous set of values such as the score of interaction between molecules. These continuous values can be converted into a classification problem by binning the scores into ranges that indicate whether the interaction is favorable or unfavorable for synergy.^79,80^

There are various strategies to design a ML model to predict molecular interactions and classify them as synergistic or not.^79^ A logical approach adopted by Combination Synergy Estimation (CoSynE)^81^ and Network-based Laplacian Regularized Least Square Synergistic Drug Combination Prediction (NLLSS)^82^ leverages drug information, including structure and confirmed interactions, to train a ML model for these predictions. The advantage of this method is the widespread availability of such data for most of the antibiotic molecules. However, this approach cannot predict the mechanisms of action underlying the synergism.^79^

Recently,^83^ researchers focused on understanding the interplay between conformational flexibility and aggregation in synergistic AMPs.^83^ This introduces a computational method that combines molecular dynamics simulations and unsupervised ML to isolate and characterize the conformations of AMPs, specifically, the WF1a and WF2 peptides. The study investigates how mixing WF1a and WF2 AMPs influences their aggregation behavior, leading to the formation of higher-order aggregates. By utilizing unsupervised learning and molecular dynamics simulations, the authors demonstrate that combining the WF1a and WF2 peptides restricts their conformational space, reducing the number of distinct conformations adopted by the peptides, especially for WF2.^83^ The findings shed light on how the interaction between the WF1a and WF2 peptides modulates the distribution of WF2 conformations within aggregates, providing insights into how one peptide can influence the behavior of another in a synergistic manner. Overall, the paper contributes to a deeper understanding of the synergy between AMPs, offering valuable insights into their structural dynamics, aggregation behavior, and potential implications for developing novel antimicrobial strategies.^83^

In another recent study, a ML algorithm was developed using supervised learning techniques to predict the FIC index of AMPs and antimicrobial agents. This study marks the first successful attempt to predict these interactions accurately. Leveraging data from the DBAASP and DrugBank databases, the algorithm evaluated a diverse set of variables. The hyperparameter-optimized light-gradient-booted machine classifier (oLGBMC) achieved a notable test accuracy of 76.92% in predicting synergistic effects. Feature importance analysis indicated that key points for predicting synergistic effects included the target microbial species, MICs of AMPs and antimicrobial agents, and the specific antimicrobial agent used. These findings suggest that ML models can effectively forecast synergistic activities among various antimicrobial agents, potentially reducing the need for labor-intensive experimental procedures and cutting down on research costs.^78^

## 5 Conclusion and Future Prospects

AMPs, in combination with antibiotics, have become a promising strategy for countering MDR bacterial infections. As discussed here, some studies have reported positive results using this combination therapy. Given that the AMP–antibiotic combination can exhibit strong antimicrobial effects at low concentrations and may reduce side effects, this therapy can also reduce production and hospital costs. However, AMPs in combination with antibiotics have sometimes been ineffective *in vitro* and *in vivo*. Such variations in a single therapeutic strategy reveal that there is still much to be investigated regarding the rules that govern AMP–antibiotic synergism. Therefore, further efforts are needed to modulate the appropriate therapeutic doses of AMPs and antibiotics. Additionally, a deep understanding of these antimicrobial mechanisms of action when administered alone or in synergism will significantly contribute to identifying crucial determinants for successful combination therapies. Additionally, the prediction of synergism by computational methods helps us in understanding and selecting drugs with better effects and in facilitating data analysis to quantify synergism. Finally, considering that AMPs and antibiotics interact differently within a host organism, greater efforts are encouraged in drug-delivery systems to enhance their therapeutic effects.
